# Why do tuberculosis suspects bypass local services to attend tuberculosis sanatorium?

**DOI:** 10.4103/0970-2113.68304

**Published:** 2010

**Authors:** Rajeswari Ramachandran, M Muniyandi, P G Gopi, Fraser Wares

**Affiliations:** *Department of Clinical Research, Tuberculosis Research Centre, Indian Council of Medical Research, Chennai, India*; 1 *World Health Organisation, New Delhi, India*

**Keywords:** DOTS, India, RNTCP, sanatorium, tuberculosis, utilization

## Abstract

**Background:**

The Government Hospital of Thoracic Medicine (GHTM), Tambaram, in Kanchipuram district (formerly known as tuberculosis [TB] sanatorium), Tamil Nadu, draws patients from all over India although RNTCP services have been in place country-wide for a number of years.

**Objective:**

To document the reasons for patients with chest symptoms attending GHTM, Tambaram.

**Materials and Methods:**

In a prospective observational study, on a simple random sample basis, TB suspects attending the out-patient department of GHTM during the period January-March, 2006, were interviewed using a semi-structured interview schedule. Information on demographic, socio-economic characteristics and reasons for attending GHTM for health care was collected.

**Results:**

A total of 2,023 respondents attended GHTM during the study period; 56% were males, 67% were aged <45 years, 63% were literates and the average annual family income was Rs 25,000. Multiple reasons for attending GHTM were given: popularity of the centre (82%), perceived availability of good treatment (52%), referral by earlier treated patients (36%), expectation of specialized care (22%), referred by treating physicians (13%), and came for inpatient care (11%).

**Conclusion:**

Despite the availability of local RNTCP health services, many patients with chest symptoms made use of GHTM services due to the reputation of the former “TB sanatorium” in the community. The findings suggest that there is a need to improve community awareness of the availability of free diagnostic and treatment facilities locally under RNTCP.

## INTRODUCTION

The Government of India’s Revised National Tuberculosis Control Program (RNTCP), based on the internationally recommended Directly Observed Treatment Short-course (DOTS) strategy, is an effective public health strategy for the control of tuberculosis (TB) in India. In terms of the over 1.5 million patients it treats per year, RNTCP is the largest such program in the world. The RNTCP services provided are integrated with the nation-wide primary healthcare services and widely involve the community. RNTCP’s diagnostic and treatment services are decentralized and widely available through more than 12,500 designated microscopy centers and hundreds of thousands of treatment centers based both in health facilities and in the community. Thus, all TB suspects and patients via RNTCP services have ready access to a universal standard of TB care.[1]

The Government Hospital of Thoracic Medicine (GHTM), popularly known as ‘Tambaram TB Sanatorium,’ is a 750-bedded TB hospital which has been providing diagnostic and treatment services to chest symptomatics and TB patients since over five decades. It draws patients from all over India, with 60,711 new patients and 35,044 old patients seen as out-patients in 2006.[2] It was noted that although RNTCP services have been in place across the whole of Tamil Nadu state since early 2002, TB suspects from all over the state were still seeking care at GHTM and that over the recent years out-patient attendance numbers had not come down. For patients, this “bypassing” of the locally available RNTCP TB services involves traveling long distances, with resultant loss of income and time, and if admitted for in-patient care, additional expenditure is incurred.

Is this due to a lack of awareness or non-acceptability amongst patients of the RNTCP diagnostic and treatment facilities available locally and/or referral of patients to GHTM by practitioners?

To answer these questions, a study was undertaken to assess the reasons given by patients for attending GHTM, and to describe the socio-economic profile of these patients.

## MATERIALS AND METHODS

### Study area

A prospective study was conducted in GHTM in the Kanchipuram district of Tamil Nadu, situated 20kms south of the state capital, Chennai.

### Study population

From among the patients attending the out-patient department of the GHTM during the period January to March 2006, 20 male and 20 female patients on each working day were selected on a simple random sample basis. Patients were grouped depending on their place of residence. Group I included those symptomatics from Kanchipuram district itself, adjacent Tiruvallur district and Chennai city. Patients from this group had to travel a distance of 1-50 kms to reach GHTM. Group II included symptomatics from other districts of Tamil Nadu and from Chitoor district of Andhra Pradesh. These patients had to travel a distance of more than 30 kms to reach GHTM.

### Data collection

Trained Health Visitors (HVs) conducted the interviews after establishing a rapport with the TB suspects at GHTM’s out-patient department. Patients were informed in their mother tongue or a language that they understood, about the purpose of the study. Patients were told about the confidentiality of the data collected and also of their right to withdraw from the study at any time. A semi structured pre-coded interview schedule was used to collect reasons for attending GHTM. In addition, the demographic, socio-economic profile comprising education, occupation, income and information about the housing were collected.

### Data management

After scrutiny, the data were computerized and to ensure accuracy all records were keyed in twice by two independent data entry operators. Data were checked for errors and analyzed using the SPSS (8.0 version SPSS Inc, Chicago, IL) package. In univariate analysis, the Chi square test was used to compare the demographic, socio economic characteristics of patients from different districts. A *P*-value <0.05 was considered statistically significant.

## RESULTS

A total of 2,023 patients (TB suspects) attending GHTM for healthcare were interviewed. Group I included 1,625 (80%) and Group II 398 (20%). The socio-demographic profile of patients of both the groups was similar [Table 1]. Of the 2,023 patients, 1,358 (67%) were aged <45 years, 1,280 (63%) were married, 1,298 (64%) lived in a nuclear family, 1,459 (72%) lived with family size >3, and 1,273 (63%) were literate.

**Table 1:** Socio-demographic characteristics of study population

	Group I (n 1625)		Group II (n 398)		Total (n 2023)	
	No	%	No	%	No	%
Sex						
Male	902	56	222	56	1124	56
Female	723	44	176	44	899	44
Age						
<45 years	1091	67	267	67	1358	67
≥45 years	534	33	131	33	665	33
Marital status						
Unmarried	428	26	98	25	526	26
Married	1024	63	256	64	1280	63
Widow/Separated	173	11	44	11	217	11
Type of family						
Joint	620	38	105	26	725	36
Nuclear	1005	62	293	74	1298	64
Family size						
≤3	459	28	105	26	564	28
>3	1166	72	293	74	1459	72
Education						
Illiterate	584	36	166	42	750	37
Literate	1041	64	232	58	1273	63

Group I: Patients from adjacent districts, Group II: Patients from other districts

Table 2 describes the economic characteristics of respondents attending GHTM for health care. Of the 2,023 patients, 1,285 (64%) were employed, 1,705 (84%) were landless, 834 (41%) lived in *kutcha* houses (made from mud, thatch or other low-quality materials) and 757 (37%) in *semi-pucca* houses. There were 1,229 (61%) patients who lived with only one earning member in the family, 680 (34%) lived in rented house, and 1,107 (55%) had only one person in the household who had studied more than 8^th^ standard. The average (median) annual family income was Rs 25,000.

**Table 2:** Economic profile of study population

	Group I (n 1625)		Group II (n 398)		Total (n 2023)	
	No	%	No	%	No	%
Occupation						
Employed	1031	63	254	64	1285	64
Unemployed	594	37	144	36	738	36
Ownership of land						
Land less	1366	84	339	85	1705	84
1-2 acres	200	12	45	11	245	12
>2 acres	59	4	14	4	73	4
House type*						
Kutcha	668	41	166	42	834	41
Semi pucca	614	38	143	36	757	37
Pucca	343	21	89	22	432	21
Earning members						
1	992	61	237	60	1229	61
2	477	29	132	33	609	30
≥3	156	10	29	7	185	9
Ownership of house						
Rent	539	33	141	35	680	34
Own	1086	67	257	65	1343	66
No studied >8^th^ std						
1	893	55	214	54	1107	55
2	399	25	115	29	514	25
≥3	333	20	69	17	402	20
Median annual family income (Rs)	24000		26300		25000	

Group I: Patients from adjacent districts, Group II: Patients from other districts

* Kutcha = Made from mud, thatch or other low-quality materials, Semi-pucca =Using partly low-quality and partly high quality materials, Pucca = Made with high-quality materials throughout including the roof, walls and floors

Table 3 describes reasons why respondents had come to GHTM and the reasons given were multiple. Majority (1,652; 82%) of them came to GHTM due to the popularity of the centre. This popularity was significantly higher among Group I patients than Group-II (84% vs. 74%; *p*<0.001). The perception of going to get ‘good treatment’ was given by 1,047 (52%) patients (46% in Gp I vs. 75% in Gp II; p<0.001). A significant number (36%) of patients were referred by old patients of GHTM (Gp I 33% vs. Gp II 50%; p<0.001) and 28% by relatives (Gp I 27% vs. Gp II 33%; P<0.05). Neighbors referred 506 (25%) and 257 (13%) were referred by their treating physician. Other reasons given were the expectation of receiving specialized care by 440 (22%) patients and inpatient care by 229 (11%).

**Table 3:** Reasons for coming to Tambaram

	Group I(n 1625)		Group II (n 398)		Total (n 2023)	
	No	%	No	%	No	%
Popularity of sanatorium	1357	84	295	74	1652	82
Perception on good treatment	749	46	298	75	1047	52
Referred by patients treated at GHTM	540	33	198	50	738	36
Suggested by relatives	442	27	132	33	574	28
Suggested by neighbor	400	25	106	27	506	25
Expecting specialized care	352	22	88	22	440	22
Referred by treating physician	213	13	44	11	257	13
For inpatient care	190	12	39	10	229	11
Availability of medical Officers round-the-clock	10	1	9	2	19	1

## DISCUSSION

Our study showed that patients used the services of GHTM mainly due to its popularity as a TB sanatorium (82%) and referrals by previously treated patients at GHTM, relatives and neighbors. Similar observations were reported from other parts of India and elsewhere, where popularity and perception of getting good treatment appeared to be the motivating factors for higher utilization of sanatoria-like health care services.[3–5] A recent study reported that 25-41% of the annual case detection in Bangalore district came from TB sanatoria alone since the majority of chest symptomatics approached the sanatoria for TB care.[6] TB sanatoria were established earlier in India in several places, mainly in major cities, as an important part of the national infrastructure for TB control activities. These centers provide free TB diagnostic and treatment services in a hospital based in-patient setting. Their specialized nature still attracts large numbers of respiratory symptomatics. TB patients who visit these centers are not only from the districts in which they are located, but also from neighboring districts and states. The popularity of the GHTM was more among patients living in and around the centre, and this may be due to the proximity and easy accessibility.

During the study period, a total of 16,330 chest symptomatics attended various RNTCP health facilities across the district for diagnosis, and of them 1,625 (10%) attended GHTM. The reason for bypassing the locally available decentralized health services was mainly on the popularity of the sanatorium and the perception of patients of the availability of good quality care and environment like adequate rest, diet, close observation and cooperation at GHTM. This highlights the continued need to improve community awareness of the availability of quality and free diagnostic and treatment services at their local health facilities under RNTCP. The community needs also to be aware of the need to take timely action if they are ill with chest symptoms suggestive of TB disease.

The present study showed that among the referrals to GHTM the proportion of referrals by treating physicians was small (13%). In general, patients referred by physicians were either chronically ill or seriously ill and required either inpatient care and/or special investigations.

Data shows that the patients who attend government health services are generally from the low socio-economic segment of the population.[7] Our study also showed that most of the chest symptomatics attending GHTM were from the poorer sections of the community, with 84% being landless and 41% living in *kutcha* houses. For households with a low standard of living, the public sector is a crucial source of healthcare. An earlier study found that 64% of 864 patients notified to the RNTCP, were from section of the population with a low standard of living index (SLI), 32% from medium SLI and only 4% from the high SLI groups.[8] One of the goals of RNTCP is that the patient should not lose wages or incur expenditure for travel. Extra efforts have, therefore, been made to provide decentralized services for diagnosis and treatment as close as operationally possible to patient’s residence.[9] The present study showed that patients, even from the poorer sections of the community, continue to travel long distance to GHTM. This again emphasizes the need to create greater awareness in the community of the availability of quality and free services close to their residences.

### Limitations of the study

Before considering the implications of the study, it is important to note the study’s limitations. Firstly it was not possible for the sampling of respondents to be selected on a probabilistic basis from a defined population; hence a convenient sample of 40 consecutive outpatients was interviewed each day. The awareness of locally available RNTCP services and the reason of “bypassing” them were not directly questioned of the interviewees. This study focused on perceptions towards services and service utilization with respect to a single nominated illness episode.

## CONCLUSION

The main reasons for seeking care at the ‘TB sanatorium’ were popularity of the center and the perception of the availability of “good treatment” at the hospital. This suggests that awareness of the availability of decentralized quality free diagnostic and treatment services all across the country, has not yet penetrated into the wider society. Policy makers can utilize the study findings to develop strategies towards better education and for planning of wider community involvement in TB control activities. There is a need for future studies to see whether there is any change in this situation over time.
